# Salts of 4-[(benzyl­amino)­carbon­yl]-1-methyl­pyridinium and iodide anions with different cation:iodine stoichiometric ratios

**DOI:** 10.1107/S2056989021011300

**Published:** 2021-11-02

**Authors:** Vitalii V. Rudiuk, Anna M. Shaposhnyk, Vyacheslav M. Baumer, Igor A. Levandovskiy, Svitlana V. Shishkina

**Affiliations:** a Farmak JSC, 63 Kyrylivska str., Kyiv 04080, Ukraine; b Department of Organic Chemistry, National Technical University of Ukraine, 37 Pobedy ave., Kyiv 03056, Ukraine; c SSI "Institute for Single Crystals", NAS of Ukraine, 60 Nauky ave., Kharkiv 61001, Ukraine; d V.N. Karazin Kharkiv National University, 4 Svobody sq., Kharkiv 61022, Ukraine

**Keywords:** 4-[(benzyl­amino)­carbon­yl]-1-methyl­pyridinium, mol­ecular structure, crystal structure, Hirshfeld analysis

## Abstract

The two iodide salts, 4-[(benzyl­amino)­carbon­yl]-1-methyl­pyridinium iodide–iodine (2/1), C_14_H_15_N_2_O^+^·I^−^·0.5I_2_, **I**, and 4-[(benzyl­amino)­carbon­yl]-1-methyl­pyridinium triiodide, C_14_H_15_N_2_O^+^·I_3_^−^, **II**, with different cation:iodine atoms ratios were studied. Salt **I** contains one cation, one iodide anion and half of the neutral I_2_ mol­ecule in the asymmetric unit (cation:iodine atoms ratio is 1:2). Salt **II** contains two cations, one triiodide anion (I**_3_^−^)** and two half triiodide anions (cation:iodine atoms ratio is 1:3). The NH group forms N—H⋯I hydrogen bonds with the I^−^ anion in the crystal of **I** or N—H⋯O hydrogen bonds in **II** where only triiodide anions are present.

## Abstract 2

The ability of 4-[(benzyl­amino)­carbon­yl]-1-methyl­pyridinium to form iodide salts with cation:iodine ratio different from equimolar was studied and a Hirshfeld surface analysis was performed to investigate the inter­molecular inter­actions.

## Chemical context

4-[(Benzyl­amino)­carbon­yl]-1-methyl­pyridinium iodide, chemical formula C_14_H_15_N_2_O^+^·I^−^, is used as a multimodal anti­viral drug (te Velthuis *et al.*, 2020 ▸; Boltz *et al.*, 2018 ▸; Buhtiarova *et al.*, 2003 ▸; Frolov *et al.*, 2004 ▸). Its mol­ecular and crystal structure have been studied in detail by diffraction and spectroscopic methods (Drebushchak *et al.*, 2017 ▸). The formation of different polymorphic modifications of an API is of great importance for the pharmaceutical industry (Bernstein, 2002 ▸; Brittain, 2009 ▸; Hilfiker, 2006 ▸). Unfortunately, all attempts to find polymorphic modifications of 4-[(benzyl­amino)­carbon­yl]-1-methyl­pyridinium iodide resulting from varying the solvents and crystallization conditions have failed. Only one crystal form with the *P*2_1_2_1_2_1_ ortho­rhom­bic space group has been determined by single-crystal X-ray diffraction (Drebushchak *et al.*, 2017 ▸). 
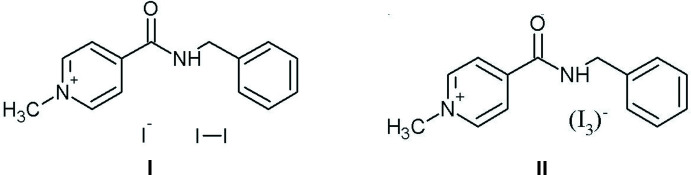


In a continuation of this work, we attempted to obtain a new polymorphic form of this compound using not only different solvents (ethanol, methanol, 2-propanol, *etc*.), but also non-standard methods of activating the crystallization process. To do this, experiments on recrystallization from water under an ultrasonic field effect were carried out. It should be noted that under normal conditions, 4-[(benzyl­amino) carbon­yl]-1-methyl­pyridinium iodide does not dissolve in water. As result, we did not obtain any new polymorphic modifications of this salt, but two compounds with cation–iodine ratios different from the equimolar [1:2 (salt **I**) and 1:3 (salt **II**)] were obtained.

## Structural commentary

**Figure 1:**
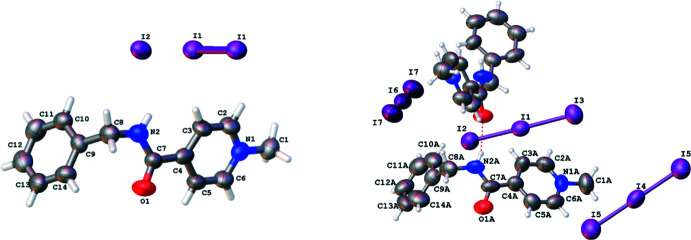
Mol­ecular structures of **I** (on the left) and **II** (on the right), showing the atom labeling scheme. Displacement ellipsoids are drawn at the 50% probability level.

The crystal structures of the salts under study consist of the same 4-[(benzyl­amino)­carbon­yl]-1-methyl­pyridinium cation (C_14_H_15_N_2_O^+^) and different anions. There is one cation, one iodide anion and half of the neutral I_2_ mol­ecule in the asymmetric unit of compound **I** (Fig. 1 ▸, left). The neutral I_2_ mol­ecule is located in a special position in relation to the symmetry centre coinciding with the midpoint of the I—I bond. Thus, the cation:iodine atoms ratio is 1:2 in compound **I**. The asymmetric unit of compound **II** contains two cations (*A* and *B*), one triiodide anion (I**_3_^−^)** and two halves of triiodide anions located on special positions in relation to the symmetry centre (Fig. 1 ▸, right). The cation:iodine atoms ration is 1:3 in compound **II**.

The positive charge of the cation is localized at the quaternized nitro­gen atom of the pyridine ring. This results in the N1—C6 and N1—C2 bond elongation (Table 1 ▸). The carbamide group is non-coplanar to the plane of the aromatic ring (as evidenced by the N2—C7—C4—C3 torsion angles; Table 1 ▸) as a result of steric repulsion between them [with short H2⋯H3 and H2⋯C3 contacts (as compared to the van der Waals radii sums; Zefirov, 1997 ▸) of 2.34 and 2.87 Å, respectively]. The cations in the two compounds under study differ in the conformation of the benzyl substituent. The phenyl fragment of the benzyl substituent is located in a −*sc* position relatively to the C7—N2 bond in **I** or in a +*sc* position in mol­ecule *A* and an *ap* position in mol­ecule *B* of **II** (*cf* the C7—N2—C8—C9 torsion angles in Table 1 ▸). The aromatic ring is turned relative to the carbamide fragment (see the N2—C8—C9—C10 torsion angles).

## Supra­molecular features

**Figure 2:**
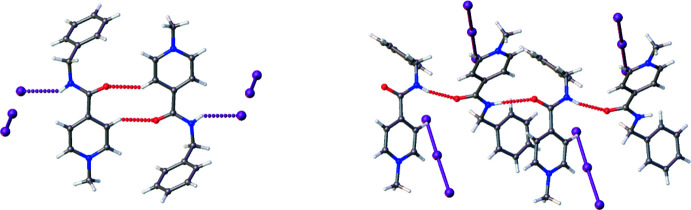
Hydrogen bond formation in structure **I** (on the left) and **II** (on the right).

The main difference in the crystal structures of the studied salts is the participation of the carbamide group in inter­molecular inter­actions. In the structure of **I**, the carbamide group participates in the N—H⋯I′ hydrogen bond between the cation and the anion, while the carbonyl oxygen atom acts as an acceptor in the very weak C5—H⋯O1′ inter­molecular inter­action (Fig. 2 ▸, left; Table 2 ▸). In the structure of **II**, the carbamide group participates in the N—H⋯O′ hydrogen bonds between the cations (Fig. 2 ▸, right; Table 3 ▸). As a result, chains in the [100] crystallographic direction are formed. The triiodide anions occupy voids between neighbouring chains in the crystal. In addition, a set of weak C—H⋯I and C—H⋯π hydrogen bonds are found in both structures (Tables 2 ▸ and 3 ▸).

In the structure of **II**, the *A* and *B* cations form stacking dimers as a result of the inter­action of the aromatic systems of the pyridine and benzene rings [the distance between the planes of aromatic cycles is 3.45 (1) Å, slippage 1.119 Å).

## Hirshfeld surface analysis

**Figure 3:**
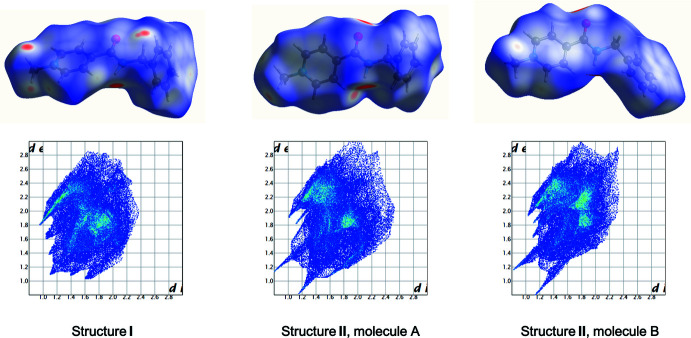
Hirshfeld surfaces mapped over *d*_norm._(at the top) and two-dimensional fingerprint plots (at the bottom) of cation in structure **I** and **II.**

Inter­molecular inter­actions can be analyzed using Hirshfeld surface analysis and 2D fingerprint plots (Turner *et al.*, 2017 ▸). The Hirshfeld surfaces were calculated for the cations found in two structures under study using a standard high surface resolution, mapped over *d*_norm_ (Fig. 3 ▸). The red spots, corresponding to contacts that are shorter than the van der Waals radii sum of the closest atoms, are observed at the hydrogen atom of the amino group. At the carbonyl group, red spots are found only in the cations of **II**. The two-dimensional fingerprint plots show that the hydrogen bonds in **II** are stronger (note the sharp spikes in Fig. 3 ▸).

**Figure 4:**
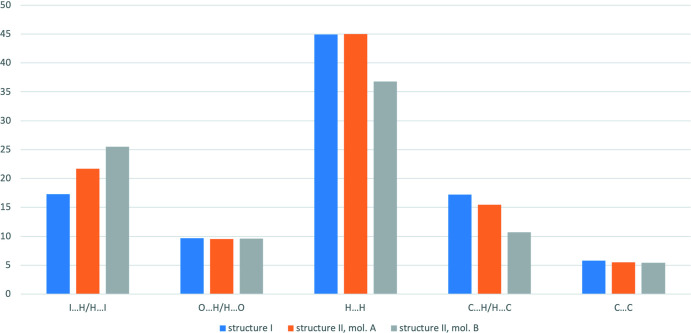
Relative contributions of the strongest inter­molecular inter­actions (in %) to the total Hirshfeld surface of cation in two iodide salts.

To compare inter­molecular inter­actions of different types in more qu­anti­tative way, their contributions to the total Hirshfeld surfaces were analysed (Fig. 4 ▸). The main contribution is provided by H⋯H short contacts (44.9% for **I**, 45% for cation *A* and 36.8% for cation *B* in **II**). The contribution of the I⋯H/H⋯I short contacts is also significant [17.3% in **I**, 21.7% (mol­ecule *A*) and 25.5% (mol­ecule *B*) in **II**], as is that of the C⋯H/H⋯C inter­actions [17.2% in **I**, 15.5% (mol­ecule *A*) and 10.7% (mol­ecule *B*) in **II**]. Surprisingly, the contributions of the O⋯H/H⋯O inter­actions are very similar in the two structures [9.7% in **I**, 9.5% (mol­ecule *A*) and 9.6% (mol­ecule *B*) in **II**] despite the stronger N—H⋯O hydrogen bonds in the structure of **II.**

## Database survey

A search of the Cambridge Structural Database (Version 5.42, update of November 2020; Groom *et al.*, 2016 ▸) revealed the structure of the AmI salt with an equimolar cation:iodine atoms ratio (refcode BEBFIA; Drebushchak *et al.*, 2017 ▸). A comparison of the cation conformations showed its flexibility resulting from rotation about the N—C*sp* ^3^ and C*sp* ^3^—Car bonds.

## Synthesis and crystallization

Benzyl­amide isonicotinic acid (124 g, 0.585 mol) and 270 mL of 90% ethanol were loaded into a glass flask. The obtained solution was heated to a temperature of 313–314 K, and then methyl iodide (91g, 0.641 mol) was added dropwise. The reaction was stirred at a temperature of 313–314 K for 1 h, heated to boiling and boiled for 1 h. The reaction spontaneously cooled to a temperature of 313 K, then to a temperature of 283–288 K in a cooling water bath, and was stirred for 1.5 h at this temperature. The reaction mixture was filtered and the precipitate rinsed on the filter twice with 60 mL of cooled 96% ethanol. The product was dried at 313 K for 12 h. Yield: 145.5 g of crude 4-[(benzyl­amino)­carbon­yl]-1-methyl­pyridinium iodide (88%); yellow crystals.

145.5 g of crude 4-[(benzyl­amino)­carbon­yl]-1-methyl­pyridinium iodide were dissolved in 450 mL of water under ultrasonic activation. The reaction was heated to boiling temperature, stirred at boiling for 30 min and filtered. The obtained solution was cooled slowly and evaporated for three weeks. The rod-shaped crystals of **I** and block-shaped crystals of **II** crystallized almost simultaneously.

## Refinement

Crystal data, data collection and structure refinement details are summarized in Table 4 ▸. Despite the presence of iodine atoms, crystals of salt **II** diffracted poorly due to their small size. All of the hydrogen atoms were located in difference-Fourier maps. Then, hydrogen atoms were refined as riding (AFIX 33 and 137 commands) with C—H = 0.96 Å, *U*_iso_(H) = 1.5*U*_eq_(C) for methyl groups (AFIX 43) and C_ar_—H = 0.93 Å, *U*_iso_(H) = 1.2*U*_eq_(C) for aromatic rings (AFIX 23) and C*sp* ^2^—H = 0.97 Å, *U*_iso_(H) = 1.2*U*_eq_(C) for the methyl­ene fragment.

## Powder diffraction characterization

**Figure 5:**
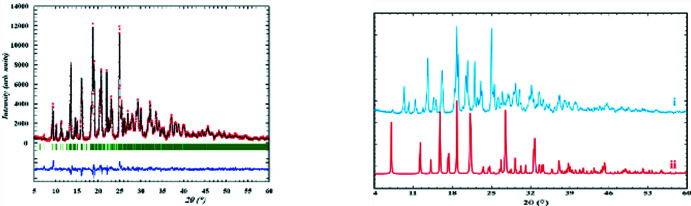
Final Rietveld plots for **II** (on the left). Observed data points are indicated by red circles, the best-fit profile (black upper trace) and the difference pattern (blue lower trace) are shown as solid lines. The vertical green bars correspond to the Bragg positions of peaks. The calculated powder pattern for **I** is shown on the right.

A powder diffraction pattern of salt **II** was registered using a Siemens D500 powder diffractometer (Cu *K*α radiation, Bragg–Brentano geometry, curved graphite monochromator on the counter arm, 4 < 2θ < 60°, *D*2θ = 0.02°, time per step of 2 s). The Rietveld refinement of the obtained pattern (Fig. 5 ▸, left) was carried out with *FULLPROF* (Rodriguez-Carvajal, 2001 ▸) and *WINPLOTR* (Roisnel & Rodriguez-Carvajal, 2000 ▸) using an external standard (NIST SRM1976) for the calculation of the instrumental profile function and the single-crystal results as the structure model for the refinement. A powder pattern for salt **I** was not obtained because of the small amount of the crystal sample. For comparison, Fig. 5 ▸ (right) shows the pattern calculated for salt **I**.

## Supplementary Material

Crystal structure: contains datablock(s) I, II. DOI: 10.1107/S2056989021011300/ex2050sup1.cif

Structure factors: contains datablock(s) I. DOI: 10.1107/S2056989021011300/ex2050Isup2.hkl

Structure factors: contains datablock(s) II. DOI: 10.1107/S2056989021011300/ex2050IIsup3.hkl

CCDC references: 2118096, 2118095

Additional supporting information:  crystallographic information; 3D view; checkCIF report

## Figures and Tables

**Table 1:** Selected geometrical parameters (Å, °) for the cations in salts **I** and **II**

Parameter	**I**	**II** *A*	**II** *B*
N1—C2	1.338 (10)	1.327 (19)	1.32 (2)
N1—C6	1.324 (11)	1.35 (2)	1.313 (18)
N2—C7—C4—C3	18.1 (13)	−16 (2)	18 (2)
C7—N2—C8—C9	−75.0 (11)	−81 (2)	178.3 (14)
N2—C8—C9—C10	−77.6 (11)	−61.6 (18)	−53 (2)
H2⋯H3	2.09	2.14	2.11
C3⋯H2	2.55	2.61	2.57

**Table 2:** Hydrogen-bond geometry (Å, °) for **I** [Chem scheme1]

*D*—H⋯*A*	*D*—H	H⋯*A*	*D*⋯*A*	*D*—H⋯*A*
N2—H2⋯I2	0.86	2.84	3.632 (7)	154
C2—H2*A*⋯I2^i^	0.93	3.18	4.053 (9)	158
C1—H1*B*⋯I2^i^	0.96	3.11	3.992 (9)	153
C1—H1*C*⋯I2^ii^	0.96	2.96	3.908 (9)	171
C1—H1*A*⋯I1^iii^	0.96	3.00	3.824 (10)	145
C5—H5⋯O1^iv^	0.93	2.59	3.328 (11)	136
C8—H8*B*⋯C11^v^	0.97	2.80	3.590 (15)	140
C8—H8*B*⋯C10^v^	0.97	2.76	3.694 (14)	162

Symmetry codes: (i) -x+1, -y+1, -z+1; (ii) x-{\script{1\over 2}}, -y+{\script{3\over 2}}, z-{\script{1\over 2}}; (iii) x-{\script{1\over 2}}, -y+{\script{1\over 2}}, z-{\script{1\over 2}}; (iv) -x, -y+1, -z+1; (v) -x+{\script{1\over 2}}, y-{\script{1\over 2}}, -z+{\script{3\over 2}}.

**Table 3:** Hydrogen-bond geometry (Å, °) for **II** [Chem scheme1]

*D*—H⋯*A*	*D*—H	H⋯*A*	*D*⋯*A*	*D*—H⋯*A*
N2*A*—H2*A*⋯O1*B*	0.86	2.02	2.846 (14)	160
C3*A*—H3*A*⋯O1*B*	0.93	2.53	3.381 (18)	152
C2*A*—H2*AA*⋯I3	0.93	3.08	3.998 (17)	169
C1*A*—H1*AC*⋯C12*A* ^i^	0.96	2.72	3.62 (2)	158
C1*A*—H1*AA*⋯I7^i^	0.96	3.09	3.966 (19)	153
N2*B*—H2*B*⋯O1*A* ^ii^	0.86	2.13	2.986 (14)	176
C3*B*—H3*B*⋯O1*A* ^ii^	0.93	2.21	3.060 (17)	151
C2*B*—H2*BA*⋯C12*A* ^iii^	0.93	2.85	3.72 (2)	156
C1*B*—H1*BB*⋯I7^iv^	0.96	3.07	3.819 (18)	136
C6*B*—H6*B*⋯I4^v^	0.93	3.12	4.019 (17)	164

Symmetry codes: (i) x, -y+{\script{3\over 2}}, z-{\script{1\over 2}}; (ii) x+1, y, z; (iii) -x+1, -y+1, -z+1; (iv) -x, -y+1, -z+1; (v) -x, y-{\script{1\over 2}}, -z+{\script{1\over 2}}.

**Table 4:** Experimental details

	**I**	**II**
Crystal data		
Chemical formula	C_14_H_15_N_2_O^+^·I^−^·0.5I_2_	C_14_H_15_N_2_O^+^·I_3_^−^
*M*_r_	481.08	608.61
Crystal system, space group	Monoclinic, *P*2_1_/*n*	Monoclinic, *P*2_1_/*c*
Temperature (K)	293	293
*a*, *b*, *c* (Å)	14.407 (3), 8.8491 (10), 14.555 (4)	9.914 (2), 27.805 (4), 14.113 (3)
β (°)	119.63 (3)	107.83 (2)
*V* (Å^3^)	1613.0 (7)	3703.4 (12)
*Z*	4	8
Radiation type	Mo *K*α	Mo *K*α
μ (mm^−1^)	3.89	5.07
Crystal size (mm)	0.60 × 0.10 × 0.05	0.03 × 0.03 × 0.02

Data collection		
Diffractometer	Xcalibur, Sapphire3	Xcalibur, Sapphire3
Absorption correction	Multi-scan (*CrysAlis PRO*; Rigaku OD, 2018 ▸)	Multi-scan (*CrysAlis PRO*; Rigaku OD, 2018 ▸)
*T*_min_, *T*_max_	0.159, 1.000	0.347, 1.000
No. of measured, independent and observed [*I* > 2σ(*I*)] reflections	11491, 3698, 1941	21040, 6496, 2548
*R*_int_	0.083	0.124
(sin θ/λ)_max_ (Å^−1^)	0.650	0.595

Refinement		
*R*[*F* ^2^ > 2σ(*F* ^2^)], *wR*(*F* ^2^), *S*	0.053, 0.157, 1.03	0.065, 0.187, 0.97
No. of reflections	3698	6496
No. of parameters	173	371
H-atom treatment	H-atom parameters constrained	H-atom parameters constrained
Δρ_max_, Δρ_min_ (e Å^−3^)	0.90, −0.90	0.70, −0.77

Computer programs: *CrysAlis PRO* (Rigaku OD, 2018 ▸), *SHELXT2014/5* (Sheldrick, 2015*a* ▸), *SHELXL2016/6* (Sheldrick, 2015*b* ▸), *Mercury* (Macrae *et al.*, 2020 ▸) and *OLEX2* (Dolomanov *et al.*, 2009 ▸).

## supplementary crystallographic information

### 4-[(Benzylamino)carbonyl]-1-methylpyridinium iodide–iodine (2/1) (I) Crystal data

**Table:**

C_14_H_15_N_2_O^+^·I^−^·0.5I_2_	*F*(000) = 908
*M**_r_* = 481.08	*D*_x_ = 1.981 Mg m^−^^3^
Monoclinic, *P*2_1_/*n*	Mo *K*α radiation, λ = 0.71073 Å
*a* = 14.407 (3) Å	Cell parameters from 928 reflections
*b* = 8.8491 (10) Å	θ = 3.6–21.8°
*c* = 14.555 (4) Å	µ = 3.89 mm^−^^1^
β = 119.63 (3)°	*T* = 293 K
*V* = 1613.0 (7) Å^3^	Stick, red
*Z* = 4	0.60 × 0.10 × 0.05 mm

### 4-[(Benzylamino)carbonyl]-1-methylpyridinium iodide–iodine (2/1) (I) Data collection

**Table:**

Xcalibur, Sapphire3 diffractometer	3698 independent reflections
Radiation source: Enhance (Mo) X-ray Source	1941 reflections with *I* > 2σ(*I*)
Detector resolution: 16.1827 pixels mm^-1^	*R*_int_ = 0.083
ω scans	θ_max_ = 27.5°, θ_min_ = 3.2°
Absorption correction: multi-scan (CrysAlisPro; Rigaku OD, 2018)	*h* = −18→18
*T*_min_ = 0.159, *T*_max_ = 1.000	*k* = −11→11
11491 measured reflections	*l* = −18→18

### 4-[(Benzylamino)carbonyl]-1-methylpyridinium iodide–iodine (2/1) (I) Refinement

**Table:**

Refinement on *F*^2^	0 restraints
Least-squares matrix: full	Hydrogen site location: inferred from neighbouring sites
*R*[*F*^2^ > 2σ(*F*^2^)] = 0.053	H-atom parameters constrained
*wR*(*F*^2^) = 0.157	*w* = 1/[σ^2^(*F*_o_^2^) + (0.0416*P*)^2^] where *P* = (*F*_o_^2^ + 2*F*_c_^2^)/3
*S* = 1.03	(Δ/σ)_max_ = 0.001
3698 reflections	Δρ_max_ = 0.90 e Å^−^^3^
173 parameters	Δρ_min_ = −0.89 e Å^−^^3^

### 4-[(Benzylamino)carbonyl]-1-methylpyridinium iodide–iodine (2/1) (I) Special details

**Table:**

Geometry. All esds (except the esd in the dihedral angle between two l.s. planes) are estimated using the full covariance matrix. The cell esds are taken into account individually in the estimation of esds in distances, angles and torsion angles; correlations between esds in cell parameters are only used when they are defined by crystal symmetry. An approximate (isotropic) treatment of cell esds is used for estimating esds involving l.s. planes.

### 4-[(Benzylamino)carbonyl]-1-methylpyridinium iodide–iodine (2/1) (I) Fractional atomic coordinates and isotropic or equivalent isotropic displacement parameters (Å^2^)

**Table:**

	*x*	*y*	*z*	*U*_iso_*/*U*_eq_	
I1	0.51630 (5)	0.11900 (6)	0.57075 (6)	0.0696 (2)	
I2	0.55433 (5)	0.39112 (7)	0.74152 (6)	0.0738 (3)	
O1	0.1044 (5)	0.3003 (8)	0.5772 (6)	0.090 (2)	
N1	0.1782 (6)	0.6060 (7)	0.3371 (6)	0.0614 (18)	
N2	0.2820 (6)	0.2698 (8)	0.6521 (6)	0.0652 (19)	
H2	0.338519	0.295372	0.650010	0.078*	
C1	0.1732 (8)	0.7074 (10)	0.2544 (8)	0.075 (3)	
H1A	0.127757	0.663816	0.186085	0.113*	
H1B	0.243679	0.720760	0.264125	0.113*	
H1C	0.145051	0.803613	0.259012	0.113*	
C2	0.2636 (7)	0.5182 (10)	0.3929 (8)	0.069 (3)	
H2A	0.320082	0.521097	0.379074	0.083*	
C3	0.2692 (7)	0.4235 (10)	0.4707 (8)	0.067 (2)	
H3	0.327838	0.360259	0.506976	0.081*	
C4	0.1885 (6)	0.4224 (9)	0.4944 (8)	0.062 (2)	
C5	0.1012 (7)	0.5127 (12)	0.4336 (8)	0.078 (3)	
H5	0.043510	0.511632	0.445534	0.093*	
C6	0.0979 (7)	0.6028 (10)	0.3569 (8)	0.070 (3)	
H6	0.038316	0.663303	0.317556	0.084*	
C7	0.1885 (6)	0.3260 (10)	0.5791 (7)	0.057 (2)	
C8	0.2932 (8)	0.1666 (10)	0.7357 (8)	0.073 (3)	
H8A	0.362531	0.117925	0.766315	0.087*	
H8B	0.239142	0.088488	0.704706	0.087*	
C9	0.2828 (7)	0.2439 (8)	0.8213 (7)	0.057 (2)	
C10	0.3668 (7)	0.3281 (10)	0.8984 (8)	0.068 (2)	
H10	0.431231	0.333813	0.898376	0.082*	
C11	0.3548 (9)	0.4014 (10)	0.9732 (9)	0.081 (3)	
H11	0.410137	0.461576	1.022065	0.097*	
C12	0.2622 (9)	0.3890 (10)	0.9788 (9)	0.078 (3)	
H12	0.255082	0.439378	1.031040	0.094*	
C13	0.1810 (9)	0.3010 (13)	0.9058 (9)	0.083 (3)	
H13	0.118664	0.289920	0.909431	0.099*	
C14	0.1904 (7)	0.2294 (10)	0.8280 (9)	0.071 (3)	
H14	0.134482	0.170291	0.778882	0.085*	

### 4-[(Benzylamino)carbonyl]-1-methylpyridinium iodide–iodine (2/1) (I) Atomic displacement parameters (Å^2^)

**Table:**

	*U*^11^	*U*^22^	*U*^33^	*U*^12^	*U*^13^	*U*^23^
I1	0.0651 (4)	0.0655 (4)	0.0790 (5)	0.0033 (3)	0.0364 (4)	−0.0008 (3)
I2	0.0669 (4)	0.0799 (4)	0.0809 (5)	−0.0121 (3)	0.0413 (4)	−0.0181 (3)
O1	0.050 (3)	0.118 (5)	0.100 (6)	−0.008 (3)	0.036 (4)	0.012 (5)
N1	0.060 (4)	0.060 (4)	0.056 (5)	0.006 (3)	0.023 (4)	−0.002 (3)
N2	0.058 (4)	0.068 (4)	0.074 (6)	−0.007 (3)	0.036 (4)	0.003 (4)
C1	0.081 (6)	0.070 (6)	0.062 (7)	0.008 (5)	0.025 (5)	0.008 (5)
C2	0.066 (6)	0.070 (6)	0.080 (8)	0.013 (4)	0.042 (6)	−0.002 (5)
C3	0.057 (5)	0.083 (6)	0.073 (7)	0.018 (4)	0.040 (5)	0.006 (5)
C4	0.051 (5)	0.066 (5)	0.060 (6)	0.003 (4)	0.021 (4)	−0.019 (4)
C5	0.048 (5)	0.121 (8)	0.067 (7)	0.008 (5)	0.030 (5)	0.004 (6)
C6	0.059 (5)	0.075 (6)	0.073 (7)	0.017 (4)	0.031 (5)	0.005 (5)
C7	0.050 (4)	0.063 (5)	0.060 (6)	−0.006 (4)	0.029 (4)	−0.002 (4)
C8	0.075 (6)	0.066 (5)	0.077 (7)	0.001 (5)	0.037 (6)	0.013 (5)
C9	0.067 (5)	0.048 (4)	0.060 (6)	0.005 (4)	0.035 (5)	0.006 (4)
C10	0.059 (5)	0.072 (6)	0.072 (7)	−0.004 (4)	0.033 (5)	0.010 (5)
C11	0.086 (7)	0.068 (6)	0.074 (8)	−0.012 (5)	0.028 (6)	−0.002 (5)
C12	0.092 (8)	0.072 (6)	0.073 (8)	0.027 (5)	0.043 (7)	0.017 (5)
C13	0.075 (6)	0.106 (8)	0.070 (7)	0.014 (6)	0.039 (6)	0.019 (6)
C14	0.053 (5)	0.075 (6)	0.083 (8)	−0.003 (4)	0.032 (5)	0.016 (5)

### 4-[(Benzylamino)carbonyl]-1-methylpyridinium iodide–iodine (2/1) (I) Geometric parameters (Å, º)

**Table:**

I1—I1^i^	2.8182 (13)	C5—C6	1.353 (13)
O1—C7	1.221 (9)	C5—H5	0.9300
N1—C6	1.324 (11)	C6—H6	0.9300
N1—C2	1.338 (10)	C8—C9	1.494 (12)
N1—C1	1.475 (11)	C8—H8A	0.9700
N2—C7	1.332 (11)	C8—H8B	0.9700
N2—C8	1.465 (11)	C9—C14	1.387 (11)
N2—H2	0.8600	C9—C10	1.391 (12)
C1—H1A	0.9600	C10—C11	1.350 (14)
C1—H1B	0.9600	C10—H10	0.9300
C1—H1C	0.9600	C11—C12	1.381 (14)
C2—C3	1.378 (12)	C11—H11	0.9300
C2—H2A	0.9300	C12—C13	1.369 (15)
C3—C4	1.366 (11)	C12—H12	0.9300
C3—H3	0.9300	C13—C14	1.362 (14)
C4—C5	1.380 (12)	C13—H13	0.9300
C4—C7	1.499 (13)	C14—H14	0.9300
			
C6—N1—C2	119.8 (8)	O1—C7—N2	123.3 (8)
C6—N1—C1	119.7 (7)	O1—C7—C4	119.4 (8)
C2—N1—C1	120.5 (8)	N2—C7—C4	117.2 (7)
C7—N2—C8	123.3 (7)	N2—C8—C9	113.1 (7)
C7—N2—H2	118.4	N2—C8—H8A	109.0
C8—N2—H2	118.4	C9—C8—H8A	109.0
N1—C1—H1A	109.5	N2—C8—H8B	109.0
N1—C1—H1B	109.5	C9—C8—H8B	109.0
H1A—C1—H1B	109.5	H8A—C8—H8B	107.8
N1—C1—H1C	109.5	C14—C9—C10	118.4 (9)
H1A—C1—H1C	109.5	C14—C9—C8	120.8 (9)
H1B—C1—H1C	109.5	C10—C9—C8	120.8 (8)
N1—C2—C3	120.9 (8)	C11—C10—C9	120.0 (9)
N1—C2—H2A	119.5	C11—C10—H10	120.0
C3—C2—H2A	119.5	C9—C10—H10	120.0
C4—C3—C2	120.0 (8)	C10—C11—C12	121.5 (10)
C4—C3—H3	120.0	C10—C11—H11	119.3
C2—C3—H3	120.0	C12—C11—H11	119.3
C3—C4—C5	116.9 (9)	C13—C12—C11	118.7 (10)
C3—C4—C7	123.9 (8)	C13—C12—H12	120.7
C5—C4—C7	119.1 (8)	C11—C12—H12	120.7
C6—C5—C4	121.4 (8)	C14—C13—C12	120.7 (10)
C6—C5—H5	119.3	C14—C13—H13	119.6
C4—C5—H5	119.3	C12—C13—H13	119.6
N1—C6—C5	120.9 (8)	C13—C14—C9	120.6 (10)
N1—C6—H6	119.6	C13—C14—H14	119.7
C5—C6—H6	119.6	C9—C14—H14	119.7
			
C6—N1—C2—C3	0.4 (14)	C3—C4—C7—N2	18.1 (13)
C1—N1—C2—C3	179.7 (9)	C5—C4—C7—N2	−164.0 (9)
N1—C2—C3—C4	−2.5 (14)	C7—N2—C8—C9	−75.0 (11)
C2—C3—C4—C5	3.5 (13)	N2—C8—C9—C14	104.6 (9)
C2—C3—C4—C7	−178.6 (9)	N2—C8—C9—C10	−77.6 (11)
C3—C4—C5—C6	−2.7 (14)	C14—C9—C10—C11	−4.3 (13)
C7—C4—C5—C6	179.3 (9)	C8—C9—C10—C11	177.8 (9)
C2—N1—C6—C5	0.5 (14)	C9—C10—C11—C12	3.4 (15)
C1—N1—C6—C5	−178.9 (9)	C10—C11—C12—C13	−0.5 (15)
C4—C5—C6—N1	0.7 (16)	C11—C12—C13—C14	−1.3 (15)
C8—N2—C7—O1	2.3 (14)	C12—C13—C14—C9	0.2 (15)
C8—N2—C7—C4	−176.2 (8)	C10—C9—C14—C13	2.6 (13)
C3—C4—C7—O1	−160.5 (9)	C8—C9—C14—C13	−179.5 (9)
C5—C4—C7—O1	17.4 (13)		

Symmetry code: (i) −*x*+1, −*y*, −*z*+1.

### 4-[(Benzylamino)carbonyl]-1-methylpyridinium iodide–iodine (2/1) (I) Hydrogen-bond geometry (Å, º)

**Table:**

*D*—H···*A*	*D*—H	H···*A*	*D*···*A*	*D*—H···*A*
N2—H2···I2	0.86	2.84	3.632 (7)	154
C2—H2*A*···I2^ii^	0.93	3.18	4.053 (9)	158
C1—H1*B*···I2^ii^	0.96	3.11	3.992 (9)	153
C1—H1*C*···I2^iii^	0.96	2.96	3.908 (9)	171
C1—H1*A*···I1^iv^	0.96	3.00	3.824 (10)	145
C5—H5···O1^v^	0.93	2.59	3.328 (11)	136
C8—H8*B*···C11^vi^	0.97	2.80	3.590 (15)	140
C8—H8*B*···C10^vi^	0.97	2.76	3.694 (14)	162

Symmetry codes: (ii) −*x*+1, −*y*+1, −*z*+1; (iii) *x*−1/2, −*y*+3/2, *z*−1/2; (iv) *x*−1/2, −*y*+1/2, *z*−1/2; (v) −*x*, −*y*+1, −*z*+1; (vi) −*x*+1/2, *y*−1/2, −*z*+3/2.

### 4-[(Benzylamino)carbonyl]-1-methylpyridinium triiodide (II) Crystal data

**Table:**

C_14_H_15_N_2_O^+^·I_3_^−^	*F*(000) = 2242
*M**_r_* = 608.61	*D*_x_ = 2.183 Mg m^−^^3^
Monoclinic, *P*2_1_/*c*	Mo *K*α radiation, λ = 0.71073 Å
*a* = 9.914 (2) Å	Cell parameters from 1078 reflections
*b* = 27.805 (4) Å	θ = 3.1–18.1°
*c* = 14.113 (3) Å	µ = 5.07 mm^−^^1^
β = 107.83 (2)°	*T* = 293 K
*V* = 3703.4 (12) Å^3^	Block, yellow
*Z* = 8	0.03 × 0.03 × 0.02 mm

### 4-[(Benzylamino)carbonyl]-1-methylpyridinium triiodide (II) Data collection

**Table:**

Xcalibur, Sapphire3 diffractometer	6496 independent reflections
Radiation source: Enhance (Mo) X-ray Source	2548 reflections with *I* > 2σ(*I*)
Detector resolution: 16.1827 pixels mm^-1^	*R*_int_ = 0.124
ω scans	θ_max_ = 25.0°, θ_min_ = 3.0°
Absorption correction: multi-scan (CrysAlisPro; Rigaku OD, 2018)	*h* = −8→11
*T*_min_ = 0.347, *T*_max_ = 1.000	*k* = −33→33
21040 measured reflections	*l* = −16→16

### 4-[(Benzylamino)carbonyl]-1-methylpyridinium triiodide (II) Refinement

**Table:**

Refinement on *F*^2^	0 restraints
Least-squares matrix: full	Hydrogen site location: inferred from neighbouring sites
*R*[*F*^2^ > 2σ(*F*^2^)] = 0.065	H-atom parameters constrained
*wR*(*F*^2^) = 0.187	*w* = 1/[σ^2^(*F*_o_^2^) + (0.0424*P*)^2^] where *P* = (*F*_o_^2^ + 2*F*_c_^2^)/3
*S* = 0.97	(Δ/σ)_max_ < 0.001
6496 reflections	Δρ_max_ = 0.70 e Å^−^^3^
371 parameters	Δρ_min_ = −0.77 e Å^−^^3^

### 4-[(Benzylamino)carbonyl]-1-methylpyridinium triiodide (II) Special details

**Table:**

Geometry. All esds (except the esd in the dihedral angle between two l.s. planes) are estimated using the full covariance matrix. The cell esds are taken into account individually in the estimation of esds in distances, angles and torsion angles; correlations between esds in cell parameters are only used when they are defined by crystal symmetry. An approximate (isotropic) treatment of cell esds is used for estimating esds involving l.s. planes.

### 4-[(Benzylamino)carbonyl]-1-methylpyridinium triiodide (II) Fractional atomic coordinates and isotropic or equivalent isotropic displacement parameters (Å^2^)

**Table:**

	*x*	*y*	*z*	*U*_iso_*/*U*_eq_	Occ. (<1)
I1	0.45921 (12)	0.79328 (4)	0.65364 (9)	0.0868 (4)	
I2	0.46503 (14)	0.71598 (5)	0.78702 (11)	0.1072 (4)	
I3	0.45434 (15)	0.87375 (4)	0.50883 (10)	0.1061 (5)	
I4	0.000000	1.000000	0.500000	0.1048 (6)	
I5	−0.09620 (18)	0.93095 (5)	0.62111 (13)	0.1313 (6)	
I6	−0.4785 (8)	0.5112 (2)	0.5262 (5)	0.130 (2)	0.5
I7	−0.3252 (7)	0.5746 (2)	0.6849 (5)	0.1504 (17)	0.5
I7A	−0.3531 (7)	0.5527 (2)	0.6302 (5)	0.1504 (17)	0.5
O1A	−0.1281 (11)	0.6399 (4)	0.3910 (8)	0.092 (3)	
N1A	0.0042 (18)	0.8083 (4)	0.3781 (10)	0.081 (4)	
N2A	0.0997 (12)	0.6306 (4)	0.4111 (9)	0.078 (4)	
H2A	0.175586	0.643198	0.404694	0.094*	
C1A	0.004 (2)	0.8621 (5)	0.3785 (13)	0.110 (6)	
H1AA	−0.091508	0.873532	0.349446	0.165*	
H1AB	0.039744	0.873527	0.445762	0.165*	
H1AC	0.062284	0.873811	0.340557	0.165*	
C2A	0.1245 (19)	0.7843 (6)	0.4148 (13)	0.096 (5)	
H2AA	0.209343	0.801051	0.438759	0.115*	
C3A	0.1251 (16)	0.7345 (6)	0.4178 (12)	0.088 (5)	
H3A	0.210205	0.717786	0.440984	0.106*	
C4A	0.0012 (16)	0.7105 (6)	0.3867 (12)	0.079 (4)	
C5A	−0.1202 (19)	0.7349 (6)	0.3482 (12)	0.089 (5)	
H5A	−0.205655	0.718459	0.324995	0.107*	
C6A	−0.1183 (19)	0.7837 (7)	0.3431 (13)	0.095 (5)	
H6A	−0.202505	0.800379	0.315060	0.114*	
C7A	−0.0134 (16)	0.6579 (6)	0.3940 (11)	0.079 (4)	
C8A	0.1044 (17)	0.5796 (5)	0.4403 (13)	0.092 (5)	
H8AA	0.181584	0.564057	0.423495	0.110*	
H8AB	0.016959	0.564255	0.401688	0.110*	
C9A	0.1238 (18)	0.5715 (5)	0.5504 (12)	0.074 (4)	
C10A	0.252 (2)	0.5902 (6)	0.6130 (16)	0.097 (6)	
H10A	0.316106	0.605499	0.587126	0.117*	
C11A	0.279 (2)	0.5848 (6)	0.7157 (17)	0.106 (6)	
H11A	0.360108	0.597669	0.760162	0.128*	
C12A	0.184 (2)	0.5606 (7)	0.7493 (16)	0.106 (6)	
H12A	0.203600	0.554933	0.817130	0.127*	
C13A	0.060 (2)	0.5444 (6)	0.685 (2)	0.107 (7)	
H13A	−0.005324	0.529527	0.710907	0.128*	
C14A	0.027 (2)	0.5491 (7)	0.5838 (17)	0.121 (7)	
H14A	−0.057924	0.537576	0.540700	0.145*	
O1B	0.3841 (10)	0.6543 (4)	0.4242 (8)	0.086 (3)	
N1B	0.4543 (16)	0.5322 (5)	0.1922 (12)	0.088 (4)	
N2B	0.6125 (12)	0.6628 (4)	0.4475 (8)	0.076 (4)	
H2B	0.684595	0.655003	0.429131	0.091*	
C1B	0.4388 (19)	0.4928 (6)	0.1164 (14)	0.108 (6)	
H1BA	0.530747	0.480708	0.119642	0.162*	
H1BB	0.382401	0.467282	0.130134	0.162*	
H1BC	0.393375	0.505462	0.051088	0.162*	
C2B	0.581 (2)	0.5433 (6)	0.2527 (16)	0.102 (6)	
H2BA	0.658994	0.525293	0.250589	0.123*	
C3B	0.5997 (15)	0.5805 (5)	0.3180 (12)	0.074 (4)	
H3B	0.690985	0.589253	0.355599	0.088*	
C4B	0.4836 (14)	0.6059 (5)	0.3296 (11)	0.069 (4)	
C5B	0.356 (2)	0.5904 (6)	0.2686 (12)	0.089 (5)	
H5B	0.273994	0.605203	0.272963	0.107*	
C6B	0.3436 (18)	0.5548 (6)	0.2030 (12)	0.090 (5)	
H6B	0.253572	0.545749	0.163528	0.107*	
C7B	0.4904 (17)	0.6428 (5)	0.4034 (11)	0.071 (4)	
C8B	0.6312 (18)	0.6988 (5)	0.5285 (12)	0.085 (5)	
H8BA	0.569928	0.726128	0.502782	0.102*	
H8BB	0.601106	0.684595	0.581491	0.102*	
C9B	0.7765 (16)	0.7161 (6)	0.5702 (11)	0.072 (4)	
C10B	0.8890 (17)	0.6853 (6)	0.6049 (12)	0.084 (4)	
H10B	0.872179	0.652312	0.601274	0.100*	
C11B	1.024 (2)	0.7013 (7)	0.6443 (13)	0.097 (5)	
H11B	1.097443	0.679375	0.668821	0.116*	
C12B	1.0525 (19)	0.7495 (8)	0.6480 (11)	0.094 (5)	
H12B	1.144660	0.760430	0.676679	0.112*	
C13B	0.946 (2)	0.7812 (7)	0.6098 (13)	0.096 (5)	
H13B	0.966127	0.813824	0.608168	0.115*	
C14B	0.8040 (16)	0.7646 (6)	0.5720 (11)	0.081 (5)	
H14B	0.730116	0.786363	0.548735	0.097*	

### 4-[(Benzylamino)carbonyl]-1-methylpyridinium triiodide (II) Atomic displacement parameters (Å^2^)

**Table:**

	*U*^11^	*U*^22^	*U*^33^	*U*^12^	*U*^13^	*U*^23^
I1	0.0752 (7)	0.0882 (8)	0.0963 (8)	−0.0041 (6)	0.0254 (6)	−0.0169 (6)
I2	0.0868 (9)	0.0985 (9)	0.1316 (11)	−0.0025 (7)	0.0264 (8)	0.0075 (8)
I3	0.1210 (11)	0.0961 (9)	0.0995 (9)	−0.0168 (8)	0.0312 (8)	−0.0034 (7)
I4	0.0852 (12)	0.0960 (13)	0.1177 (14)	0.0118 (10)	0.0082 (10)	−0.0004 (10)
I5	0.1384 (14)	0.1108 (11)	0.1504 (14)	0.0019 (10)	0.0526 (12)	0.0023 (9)
I6	0.099 (4)	0.131 (5)	0.180 (7)	0.027 (3)	0.072 (5)	0.077 (4)
I7	0.117 (3)	0.148 (4)	0.200 (6)	−0.001 (3)	0.069 (4)	0.048 (3)
I7A	0.117 (3)	0.148 (4)	0.200 (6)	−0.001 (3)	0.069 (4)	0.048 (3)
O1A	0.068 (7)	0.091 (8)	0.123 (10)	−0.009 (6)	0.039 (7)	−0.023 (6)
N1A	0.106 (11)	0.062 (8)	0.085 (9)	0.004 (8)	0.043 (9)	0.012 (7)
N2A	0.048 (7)	0.080 (9)	0.105 (10)	−0.015 (7)	0.022 (7)	−0.018 (7)
C1A	0.137 (18)	0.073 (11)	0.116 (15)	−0.004 (11)	0.031 (13)	0.013 (10)
C2A	0.071 (12)	0.091 (13)	0.124 (15)	−0.013 (10)	0.029 (11)	0.002 (11)
C3A	0.058 (10)	0.079 (11)	0.115 (14)	−0.002 (9)	0.008 (9)	0.013 (9)
C4A	0.048 (9)	0.093 (12)	0.086 (11)	−0.011 (9)	0.005 (8)	−0.001 (9)
C5A	0.085 (13)	0.089 (13)	0.087 (12)	−0.024 (11)	0.018 (10)	−0.002 (9)
C6A	0.069 (11)	0.121 (16)	0.098 (13)	0.017 (12)	0.031 (10)	0.017 (11)
C7A	0.050 (9)	0.110 (14)	0.076 (11)	−0.004 (10)	0.021 (8)	−0.010 (9)
C8A	0.078 (12)	0.075 (11)	0.125 (16)	−0.011 (9)	0.035 (11)	−0.017 (10)
C9A	0.080 (11)	0.064 (10)	0.067 (11)	−0.006 (8)	0.009 (9)	−0.018 (8)
C10A	0.108 (15)	0.081 (12)	0.122 (16)	−0.001 (11)	0.063 (14)	−0.017 (11)
C11A	0.087 (14)	0.105 (15)	0.125 (18)	−0.001 (11)	0.029 (13)	−0.021 (12)
C12A	0.094 (15)	0.113 (16)	0.112 (16)	0.024 (13)	0.034 (14)	0.002 (12)
C13A	0.114 (17)	0.074 (12)	0.16 (2)	0.003 (12)	0.084 (17)	0.024 (13)
C14A	0.125 (18)	0.140 (18)	0.120 (19)	−0.011 (15)	0.072 (16)	−0.011 (14)
O1B	0.058 (7)	0.107 (8)	0.098 (8)	−0.007 (6)	0.029 (6)	−0.016 (6)
N1B	0.087 (10)	0.073 (9)	0.120 (12)	0.009 (8)	0.058 (10)	−0.002 (8)
N2B	0.042 (7)	0.105 (10)	0.078 (9)	−0.011 (7)	0.014 (6)	−0.018 (7)
C1B	0.105 (15)	0.101 (13)	0.120 (15)	−0.017 (11)	0.036 (13)	−0.017 (12)
C2B	0.075 (13)	0.060 (11)	0.18 (2)	−0.006 (10)	0.048 (14)	0.007 (12)
C3B	0.056 (9)	0.054 (9)	0.109 (13)	−0.012 (7)	0.021 (9)	−0.012 (8)
C4B	0.043 (8)	0.073 (10)	0.086 (11)	−0.010 (7)	0.011 (7)	−0.005 (8)
C5B	0.113 (15)	0.084 (12)	0.085 (12)	0.011 (11)	0.054 (12)	−0.009 (9)
C6B	0.072 (11)	0.116 (15)	0.081 (12)	−0.019 (11)	0.023 (10)	−0.007 (10)
C7B	0.073 (10)	0.075 (10)	0.077 (11)	0.015 (9)	0.038 (9)	0.005 (8)
C8B	0.094 (13)	0.075 (10)	0.086 (11)	−0.011 (9)	0.028 (10)	−0.014 (9)
C9B	0.068 (10)	0.077 (11)	0.075 (10)	−0.012 (9)	0.030 (8)	−0.018 (8)
C10B	0.066 (11)	0.089 (12)	0.089 (12)	0.011 (10)	0.014 (9)	0.003 (9)
C11B	0.077 (13)	0.125 (16)	0.092 (13)	0.005 (12)	0.030 (11)	0.003 (11)
C12B	0.072 (12)	0.140 (17)	0.064 (11)	−0.021 (13)	0.014 (9)	−0.016 (11)
C13B	0.090 (13)	0.104 (13)	0.097 (13)	−0.006 (12)	0.034 (11)	0.002 (11)
C14B	0.052 (9)	0.112 (14)	0.069 (10)	0.014 (9)	0.005 (8)	−0.014 (9)

### 4-[(Benzylamino)carbonyl]-1-methylpyridinium triiodide (II) Geometric parameters (Å, º)

**Table:**

I1—I2	2.8459 (18)	C12A—H12A	0.9300
I1—I3	3.0206 (17)	C13A—C14A	1.37 (3)
I4—I5	2.9181 (15)	C13A—H13A	0.9300
I4—I5^i^	2.9181 (15)	C14A—H14A	0.9300
I6—I6^ii^	0.962 (9)	O1B—C7B	1.220 (15)
I6—I7A	1.977 (7)	N1B—C6B	1.313 (18)
I6—I7	2.890 (7)	N1B—C2B	1.32 (2)
I6—I7A^ii^	2.925 (7)	N1B—C1B	1.504 (19)
I7—I7A	0.957 (7)	N2B—C7B	1.305 (17)
O1A—C7A	1.231 (16)	N2B—C8B	1.488 (17)
N1A—C2A	1.327 (19)	N2B—H2B	0.8600
N1A—C6A	1.35 (2)	C1B—H1BA	0.9600
N1A—C1A	1.495 (17)	C1B—H1BB	0.9600
N2A—C7A	1.315 (17)	C1B—H1BC	0.9600
N2A—C8A	1.472 (17)	C2B—C3B	1.36 (2)
N2A—H2A	0.8600	C2B—H2BA	0.9300
C1A—H1AA	0.9600	C3B—C4B	1.403 (18)
C1A—H1AB	0.9600	C3B—H3B	0.9300
C1A—H1AC	0.9600	C4B—C5B	1.36 (2)
C2A—C3A	1.39 (2)	C4B—C7B	1.447 (19)
C2A—H2AA	0.9300	C5B—C6B	1.335 (19)
C3A—C4A	1.348 (19)	C5B—H5B	0.9300
C3A—H3A	0.9300	C6B—H6B	0.9300
C4A—C5A	1.34 (2)	C8B—C9B	1.46 (2)
C4A—C7A	1.48 (2)	C8B—H8BA	0.9700
C5A—C6A	1.36 (2)	C8B—H8BB	0.9700
C5A—H5A	0.9300	C9B—C10B	1.372 (19)
C6A—H6A	0.9300	C9B—C14B	1.374 (19)
C8A—C9A	1.52 (2)	C10B—C11B	1.36 (2)
C8A—H8AA	0.9700	C10B—H10B	0.9300
C8A—H8AB	0.9700	C11B—C12B	1.37 (2)
C9A—C14A	1.35 (2)	C11B—H11B	0.9300
C9A—C10A	1.40 (2)	C12B—C13B	1.35 (2)
C10A—C11A	1.40 (2)	C12B—H12B	0.9300
C10A—H10A	0.9300	C13B—C14B	1.42 (2)
C11A—C12A	1.35 (2)	C13B—H13B	0.9300
C11A—H11A	0.9300	C14B—H14B	0.9300
C12A—C13A	1.36 (3)		
			
I2—I1—I3	178.72 (5)	C12A—C13A—C14A	122.8 (18)
I5—I4—I5^i^	180.0	C12A—C13A—H13A	118.6
I6^ii^—I6—I7A	168.1 (11)	C14A—C13A—H13A	118.6
I6^ii^—I6—I7	174.9 (11)	C9A—C14A—C13A	116 (2)
I7A—I6—I7	6.9 (3)	C9A—C14A—H14A	121.9
I6^ii^—I6—I7A^ii^	8.0 (8)	C13A—C14A—H14A	121.9
I7A—I6—I7A^ii^	176.1 (4)	C6B—N1B—C2B	118.4 (15)
I7—I6—I7A^ii^	176.9 (4)	C6B—N1B—C1B	121.6 (16)
I7A—I7—I6	14.4 (7)	C2B—N1B—C1B	120.0 (15)
I7—I7A—I6	158.7 (10)	C7B—N2B—C8B	122.2 (12)
I7—I7A—I6^ii^	162.5 (9)	C7B—N2B—H2B	118.9
I6—I7A—I6^ii^	3.9 (4)	C8B—N2B—H2B	118.9
C2A—N1A—C6A	119.2 (14)	N1B—C1B—H1BA	109.5
C2A—N1A—C1A	120.4 (16)	N1B—C1B—H1BB	109.5
C6A—N1A—C1A	120.3 (16)	H1BA—C1B—H1BB	109.5
C7A—N2A—C8A	124.0 (13)	N1B—C1B—H1BC	109.5
C7A—N2A—H2A	118.0	H1BA—C1B—H1BC	109.5
C8A—N2A—H2A	118.0	H1BB—C1B—H1BC	109.5
N1A—C1A—H1AA	109.5	N1B—C2B—C3B	121.5 (16)
N1A—C1A—H1AB	109.5	N1B—C2B—H2BA	119.3
H1AA—C1A—H1AB	109.5	C3B—C2B—H2BA	119.3
N1A—C1A—H1AC	109.5	C2B—C3B—C4B	121.0 (15)
H1AA—C1A—H1AC	109.5	C2B—C3B—H3B	119.5
H1AB—C1A—H1AC	109.5	C4B—C3B—H3B	119.5
N1A—C2A—C3A	120.7 (16)	C5B—C4B—C3B	113.7 (14)
N1A—C2A—H2AA	119.6	C5B—C4B—C7B	120.6 (14)
C3A—C2A—H2AA	119.6	C3B—C4B—C7B	125.5 (14)
C4A—C3A—C2A	119.3 (16)	C6B—C5B—C4B	122.9 (16)
C4A—C3A—H3A	120.3	C6B—C5B—H5B	118.5
C2A—C3A—H3A	120.3	C4B—C5B—H5B	118.5
C5A—C4A—C3A	119.7 (16)	N1B—C6B—C5B	122.2 (17)
C5A—C4A—C7A	115.8 (14)	N1B—C6B—H6B	118.9
C3A—C4A—C7A	124.5 (15)	C5B—C6B—H6B	118.9
C4A—C5A—C6A	120.1 (17)	O1B—C7B—N2B	121.1 (14)
C4A—C5A—H5A	120.0	O1B—C7B—C4B	120.4 (15)
C6A—C5A—H5A	120.0	N2B—C7B—C4B	118.6 (13)
N1A—C6A—C5A	120.8 (17)	C9B—C8B—N2B	113.9 (13)
N1A—C6A—H6A	119.6	C9B—C8B—H8BA	108.8
C5A—C6A—H6A	119.6	N2B—C8B—H8BA	108.8
O1A—C7A—N2A	119.8 (16)	C9B—C8B—H8BB	108.8
O1A—C7A—C4A	120.7 (14)	N2B—C8B—H8BB	108.8
N2A—C7A—C4A	119.3 (14)	H8BA—C8B—H8BB	107.7
N2A—C8A—C9A	114.4 (12)	C10B—C9B—C14B	118.1 (15)
N2A—C8A—H8AA	108.7	C10B—C9B—C8B	122.2 (15)
C9A—C8A—H8AA	108.7	C14B—C9B—C8B	119.6 (15)
N2A—C8A—H8AB	108.7	C11B—C10B—C9B	122.2 (17)
C9A—C8A—H8AB	108.7	C11B—C10B—H10B	118.9
H8AA—C8A—H8AB	107.6	C9B—C10B—H10B	118.9
C14A—C9A—C10A	123.7 (18)	C10B—C11B—C12B	120.2 (18)
C14A—C9A—C8A	123.0 (17)	C10B—C11B—H11B	119.9
C10A—C9A—C8A	113.2 (16)	C12B—C11B—H11B	119.9
C11A—C10A—C9A	117.4 (17)	C13B—C12B—C11B	119.9 (18)
C11A—C10A—H10A	121.3	C13B—C12B—H12B	120.1
C9A—C10A—H10A	121.3	C11B—C12B—H12B	120.1
C12A—C11A—C10A	119 (2)	C12B—C13B—C14B	119.9 (17)
C12A—C11A—H11A	120.5	C12B—C13B—H13B	120.1
C10A—C11A—H11A	120.5	C14B—C13B—H13B	120.1
C11A—C12A—C13A	121 (2)	C9B—C14B—C13B	119.6 (16)
C11A—C12A—H12A	119.6	C9B—C14B—H14B	120.2
C13A—C12A—H12A	119.6	C13B—C14B—H14B	120.2
			
I6—I7—I7A—I6^ii^	−2.0 (11)	C6B—N1B—C2B—C3B	−7 (3)
C6A—N1A—C2A—C3A	0 (2)	C1B—N1B—C2B—C3B	176.0 (14)
C1A—N1A—C2A—C3A	177.8 (15)	N1B—C2B—C3B—C4B	6 (3)
N1A—C2A—C3A—C4A	−3 (3)	C2B—C3B—C4B—C5B	−2 (2)
C2A—C3A—C4A—C5A	4 (3)	C2B—C3B—C4B—C7B	173.6 (15)
C2A—C3A—C4A—C7A	−174.6 (15)	C3B—C4B—C5B—C6B	−1 (2)
C3A—C4A—C5A—C6A	−2 (3)	C7B—C4B—C5B—C6B	−176.4 (14)
C7A—C4A—C5A—C6A	176.7 (14)	C2B—N1B—C6B—C5B	4 (3)
C2A—N1A—C6A—C5A	2 (2)	C1B—N1B—C6B—C5B	−178.7 (15)
C1A—N1A—C6A—C5A	−175.8 (14)	C4B—C5B—C6B—N1B	0 (3)
C4A—C5A—C6A—N1A	−1 (2)	C8B—N2B—C7B—O1B	3 (2)
C8A—N2A—C7A—O1A	−8 (2)	C8B—N2B—C7B—C4B	−176.2 (13)
C8A—N2A—C7A—C4A	166.8 (14)	C5B—C4B—C7B—O1B	13 (2)
C5A—C4A—C7A—O1A	−19 (2)	C3B—C4B—C7B—O1B	−161.8 (15)
C3A—C4A—C7A—O1A	159.5 (17)	C5B—C4B—C7B—N2B	−167.7 (14)
C5A—C4A—C7A—N2A	166.0 (15)	C3B—C4B—C7B—N2B	18 (2)
C3A—C4A—C7A—N2A	−16 (2)	C7B—N2B—C8B—C9B	178.3 (14)
C7A—N2A—C8A—C9A	−81 (2)	N2B—C8B—C9B—C10B	−53 (2)
N2A—C8A—C9A—C14A	117.5 (17)	N2B—C8B—C9B—C14B	124.6 (15)
N2A—C8A—C9A—C10A	−61.6 (18)	C14B—C9B—C10B—C11B	3 (2)
C14A—C9A—C10A—C11A	0 (3)	C8B—C9B—C10B—C11B	−179.5 (15)
C8A—C9A—C10A—C11A	179.4 (14)	C9B—C10B—C11B—C12B	−2 (3)
C9A—C10A—C11A—C12A	2 (3)	C10B—C11B—C12B—C13B	−2 (3)
C10A—C11A—C12A—C13A	−4 (3)	C11B—C12B—C13B—C14B	4 (2)
C11A—C12A—C13A—C14A	4 (3)	C10B—C9B—C14B—C13B	0 (2)
C10A—C9A—C14A—C13A	−1 (3)	C8B—C9B—C14B—C13B	−178.0 (14)
C8A—C9A—C14A—C13A	179.8 (15)	C12B—C13B—C14B—C9B	−3 (2)
C12A—C13A—C14A—C9A	−1 (3)		

Symmetry codes: (i) −*x*, −*y*+2, −*z*+1; (ii) −*x*−1, −*y*+1, −*z*+1.

### 4-[(Benzylamino)carbonyl]-1-methylpyridinium triiodide (II) Hydrogen-bond geometry (Å, º)

**Table:**

*D*—H···*A*	*D*—H	H···*A*	*D*···*A*	*D*—H···*A*
N2*A*—H2*A*···O1*B*	0.86	2.02	2.846 (14)	160
C3*A*—H3*A*···O1*B*	0.93	2.53	3.381 (18)	152
C2*A*—H2*AA*···I3	0.93	3.08	3.998 (17)	169
C1*A*—H1*AC*···C12*A*^iii^	0.96	2.72	3.62 (2)	158
C1*A*—H1*AA*···I7^iii^	0.96	3.09	3.966 (19)	153
N2*B*—H2*B*···O1*A*^iv^	0.86	2.13	2.986 (14)	176
C3*B*—H3*B*···O1*A*^iv^	0.93	2.21	3.060 (17)	151
C2*B*—H2*BA*···C12*A*^v^	0.93	2.85	3.72 (2)	156
C1*B*—H1*BB*···I7^vi^	0.96	3.07	3.819 (18)	136
C6*B*—H6*B*···I4^vii^	0.93	3.12	4.019 (17)	164

Symmetry codes: (iii) *x*, −*y*+3/2, *z*−1/2; (iv) *x*+1, *y*, *z*; (v) −*x*+1, −*y*+1, −*z*+1; (vi) −*x*, −*y*+1, −*z*+1; (vii) −*x*, *y*−1/2, −*z*+1/2.

## References

[bb1] Bernstein, J. (2002). *Polymorphism in Molecular Crystals.* Oxford: Clarendon Press.

[bb2] Boltz, D., Peng, X., Muzzio, M., Dash, P., Thomas, P. G. & Margitich, V. (2018). *Antivir. Chem. Chemother.* **26** https://doi.org/10.1177/2040206618811416. 10.1177/2040206618811416 PMC6961345 30466301

[bb3] Brittain, H. G. (2009). *Polymorphism in pharmaceutical solids*, 2nd ed. New York: Informa.

[bb4] Buhtiarova, T. A., Danilenko, V. P., Homenko, V. S., Shatyrkina, T. V. & Yadlovsky, O. E. (2003). *Ukrainian Med. J.* **33**, 72–74.

[bb5] Dolomanov, O. V., Bourhis, L. J., Gildea, R. J., Howard, J. A. K. & Puschmann, H. (2009). *J. Appl. Cryst.* **42**, 339–341.

[bb6] Drebushchak, T. N., Kryukov, Y. A., Rogova, A. I. & Boldyreva, E. V. (2017). *Acta Cryst.* E**73**, 967–970. 10.1107/S2056989017008155 PMC5499270 28775862

[bb7] Frolov, A. F., Frolov, V. M., Buhtiarova, T. A. & Danilenko, V. P. (2004). *Ukrainian Med. J.* **39**, 69–74.

[bb8] Groom, C. R., Bruno, I. J., Lightfoot, M. P. & Ward, S. C. (2016). *Acta Cryst.* B**72**, 171–179. 10.1107/S2052520616003954 PMC4822653 27048719

[bb9] Hilfiker, R. (2006). *Polymorphism in the Pharmaceutical Industry*. Weinheim: John Wiley & Sons.

[bb10] Macrae, C. F., Sovago, I., Cottrell, S. J., Galek, P. T. A., McCabe, P., Pidcock, E., Platings, M., Shields, G. P., Stevens, J. S., Towler, M. & Wood, P. A. (2020). *J. Appl. Cryst.* **53**, 226–235. 10.1107/S1600576719014092 PMC6998782 32047413

[bb11] Rigaku OD (2018). *CrysAlis PRO.* Rigaku Oxford Diffraction, Yarnton, England.

[bb12] Rodríguez-Carvajal, J. (2001). *Commission on Powder Diffraction (IUCr) Newsletter*, **26**, 12–19.

[bb13] Roisnel, T. & Rodríguez-Carvajal, J. (2000). *WinPLOTR*, *a Windows tool for powder diffraction patterns analysis*. *Mater. Sci. Forum, Proc. 7th Europ. Powder Diff. Conf. (EPDIC 7)*, edited by R. Delhez & E. J. Mittenmeijer, pp. 118–123.

[bb14] Sheldrick, G. M. (2015*a*). *Acta Cryst.* A**71**, 3–8.

[bb15] Sheldrick, G. M. (2015*b*). *Acta Cryst.* A**71**, 3–8.

[bb16] Turner, M. J., McKinnon, J. J., Wolff, S. K., Grimwood, D. J., Spackman, P. R., Jayatilaka, D. & Spackman, M. A. (2017). *CrystalExplorer17.* University of Western Australia. http://Hirshfeldsurface.net

[bb17] Velthuis, A. J. W. te, Zubkova, T. G., Shaw, M., Mehle, A., Boltz, D., Gmeinwieser, N., Stammer, H., Milde, J., Müller, L. & Margitich, V. (2020). Antimicrobial Agents and Chemotherapy, **64**, https://doi.org/10.1128/AAC.02605-20. 10.1128/AAC.02605-20 PMC8097484 33558285

[bb18] Zefirov, Yu. V. (1997). *Kristallografiya*, **42**, 936–958.

